# Heat shock protein 90 (Hsp90) inhibitor STA-9090 (Ganetespib) ameliorates inflammation in a mouse model of atopic dermatitis

**DOI:** 10.1007/s12192-023-01387-0

**Published:** 2023-10-18

**Authors:** Krzysztof Sitko, Michał Starke, Stefan Tukaj

**Affiliations:** 1 Department of Molecular Biology, Faculty of Biology, University of Gdańsk, Wita Stwosza 59, 80-308 Gdańsk, Poland; 2 Department of Plant Cytology and Embryology Faculty of Biology, University of Gdańsk, Gdańsk, Poland

**Keywords:** Atopic dermatitis, AD, Heat shock proteins 90, Hsp90, STA-9090, Ganetespib

## Abstract

Molecular chaperones belonging to the heat shock protein 90 (Hsp90) family are implicated in inflammatory processes and described as potential novel therapeutic targets in autoimmune/inflammatory skin diseases. While the pathological role of circulating Hsp90 has been recently proposed in patients with atopic dermatitis (AD), a chronic inflammatory skin disease characterized by intense itching and recurrent skin lesions, studies aimed at investigating the role of Hsp90 as a potential target of AD therapy have not yet been conducted. Here, the effects of the Hsp90 blocker STA-9090 (Ganetespib) applied systemically or topically were determined in an experimental mouse model of dinitrochlorobenzene (DNCB)-induced AD. Intraperitoneal administration of STA-9090 ameliorated clinical disease severity, histological epidermal thickness, and dermal leukocyte infiltration in AD mice which was associated with reducing the scratching behavior in DNCB-treated animals. Additionally, topically applied STA-9090 led to lowered disease activity in AD mice, reduced serum levels of IgE, and up-regulated *filaggrin* expression in lesional skin samples. Our observations suggest that Hsp90 may be a promising therapeutic target in atopic dermatitis and potentially other inflammatory or autoimmune dermatoses.

## Introduction

The classical role of the heat shock proteins (Hsps) relies on e.g., proper protein folding during translation, re-folding of denatured proteins, and stabilization of native proteins. Widely studied mammalian Hsp90 family consists of at least four members i.e., two cytosolic isoforms, such as Hsp90α and Hsp90β, as well as Grp94/gp96 and TRAP-1 isoforms localized in the endoplasmic reticulum and mitochondrion, respectively. All members of the Hsp90 family comprise a common domain structure consisting of the nucleotide (ATP)-binding domain localized in N-terminal domain, a middle domain, and the C-terminal domain (Tukaj and Węgrzyn 2016). Stress-induced Hsp90α isoform, is one of the key intra- and extracellular molecular chaperones responsible for the biological activity of numerous protein substrates (“clients”) belonging to the key signaling molecules implicated in tumorigenesis and inflammation (Seclì et al. 2021; Tukaj 2022). Recently, due to its pleiotropic activity, Hsp90 has become the subject of interest for many researchers in the context of autoimmune/inflammatory disease development (Tukaj and Węgrzyn 2016; Li et al. 2020; Bregnhøj et al. 2022; Tukaj and Sitko 2022; Ben Abdallah et al. 2023). In fact, preclinical or clinical studies revealed that using Hsp90 inhibitors with N-terminal affinity seems to be promising for the treatment of non-infectious inflammatory skin diseases (Ben Abdallah et al. 2023) including autoimmune bullous skin diseases or psoriasis due to attenuation of numerous inflammatory immune cells and signaling pathways (Kasperkiewicz et al. 2011; Tukaj et al. 2013, 2014a, 2017; Li et al. 2020; Bregnhøj et al. 2022; Rittig et al. 2023).

Atopic dermatitis (AD) is one of the most common, non-communicable, immune-mediated inflammatory skin diseases, characterized by intense itching and recurrent skin lesions. While the underlying events and key factors involved in the development and progression of AD are the subject of ongoing debate, there are at least two major and converging abnormalities in the epidermis structure due to i.e., reduced expression of filaggrin (FLG) and IgE-mediated sensitization to food and environmental allergens (Roesner and Werfel 2019; Langan et al. 2020). The abnormal structure of the epidermis leads to intensive penetration of the skin by allergens and thus to the activation of the Th2-type immune response (Guttman-Yassky et al. 2013; Varricchi et al. 2018). The involvement of innate immune cells, including mast cells and eosinophils, as well other Th subpopulations in AD, is also proven (Weidinger and Novak 2016; Czarnowicki et al. 2019).

While the pathological role of circulating Hsp90 has been recently proposed in patients with AD (Sitko et al. 2021), studies aimed at investigating the role of Hsp90 as a potential target of AD therapy have not been performed to date. Here, systemic, or local effects of the Hsp90 inhibitor STA-9090 (Ganetespib), targeting specifically ATP-binding domain of Hsp90, on development of the experimental mouse model of dinitrochlorobenzene (DNCB)-induced AD were studied.

## Material and methods

### Ethics statement

The animal study was reviewed and approved by local authorities of the Animal Care and Use Committee (Bydgoszcz, Poland).

### Disease induction

**Fig. 1:**
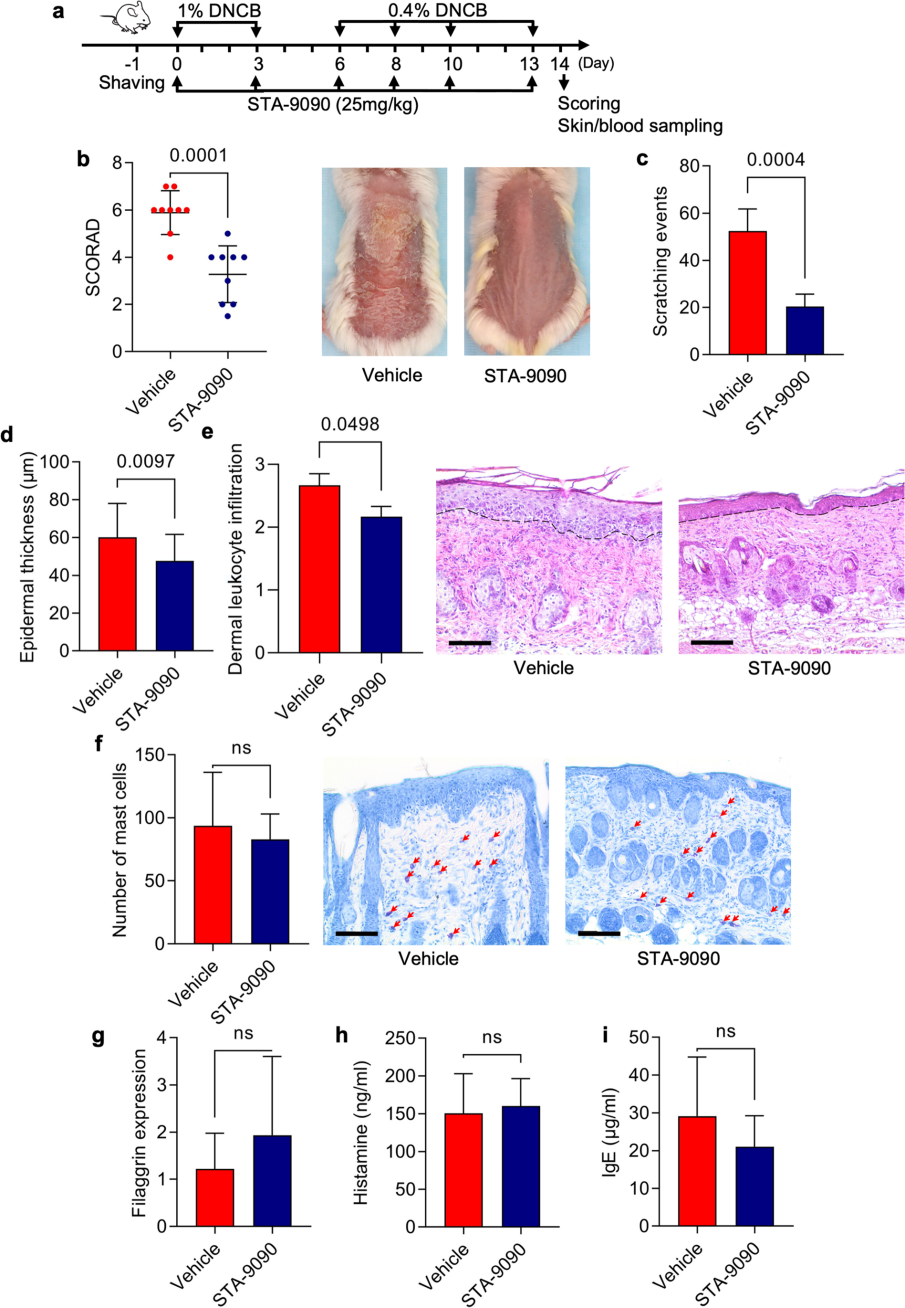
Intraperitoneal administration of Hsp90 inhibitor leads to clinical and histological disease amelioration in mice with experimental AD. (**a**) Schematic illustration of the experimental setup. AD-like skin inflammation was induced in female BALB/c mice (8-weeks old) by topical dinitrochlorobenzene (DNCB) application on shaved backs starting from day 0. Mice were treated with intraperitoneal injections of STA-9090 (25 mg/kg) in volume of 50 μL dissolved in DMSO or the equivalent volume of DMSO (Vehicle) three times a week over a 2-week treatment period starting from day 0. (**b**) Clinical disease severity of Vehicle and STA-9090-treated mice was calculated as cumulative SCORAD index at the final day (day 14) of the experiment; corresponding representative clinical presentations of vehicle- and STA-9090-treated mice at the end of the 14-day treatment period are shown on the right. (**c**) Scratching behaviour in vehicle- and STA-9090-treated animals was counted for 10 min at day 13. Histological disease severity shown by scores for (**d**) epidermal thickness, (**e**) dermal leukocyte infiltration, and (**f**) mast cells infiltration at the end of the experiment. Representative H&E and toluidine blue (red arrows indicate mast cells) staining of skin biopsies are shown next to the respective graphs. (**g**) Filaggrin (*FLG*) expression was examined in skin biopsies of vehicle- or STA-9090-treated mice at the end of the experiment by RT-qPCR. (**h**) Histamine and (**i**) IgE levels were measured in sera of vehicle- or STA-9090-treated mice at the end of the experiment by ELISA. Data are representative of three independent experiments and expressed as mean ± SEM (with individual values) of a total of 8–9 mice per group. ns, not significant. Bars = 100 μm

**Fig. 2:**
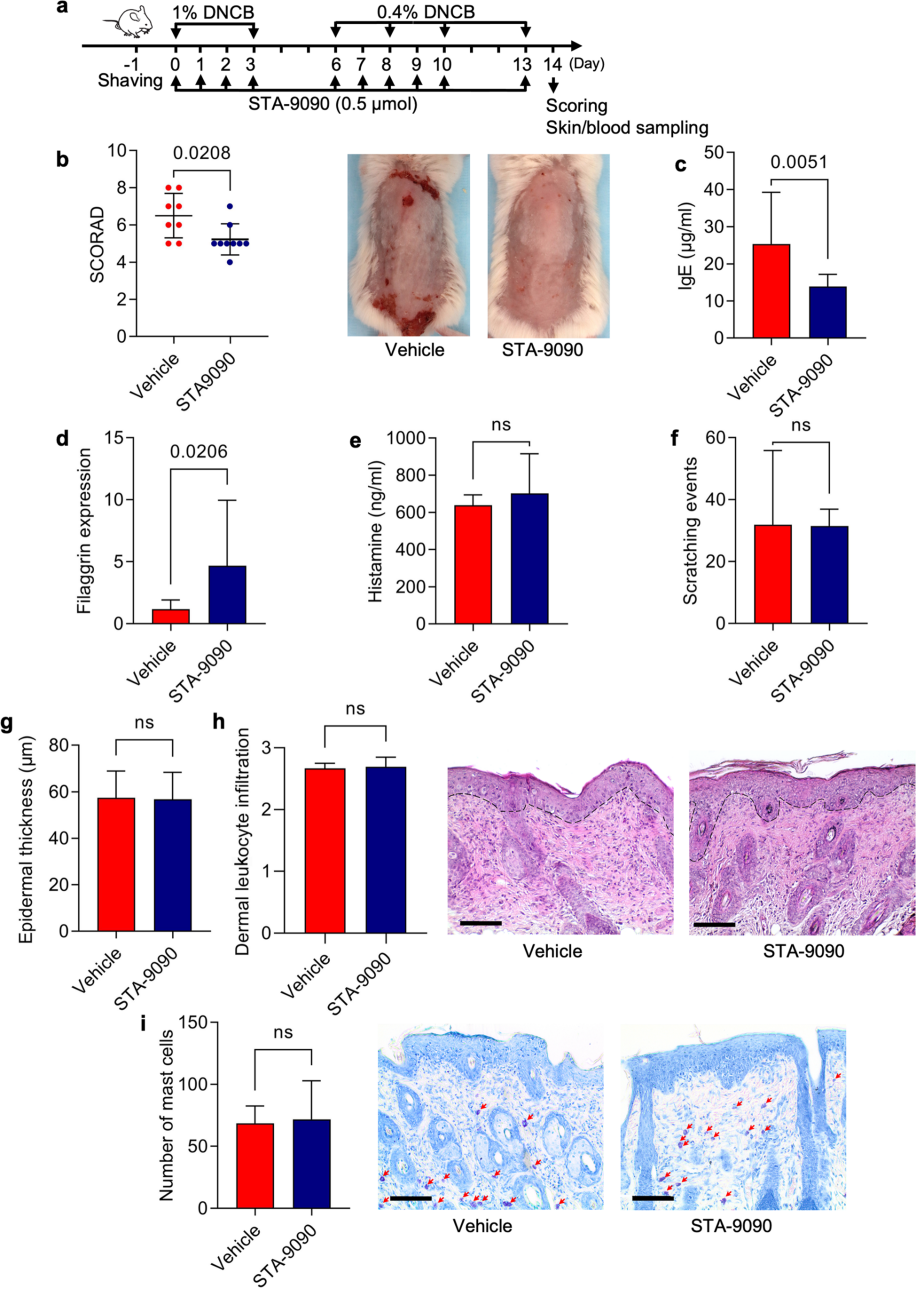
Topical administration of Hsp90 inhibitor leads to clinical and molecular disease improvement in mice with experimental AD. (**a**) Schematic illustration of the experimental setup. AD-like skin inflammation was induced in female BALB/c mice (8-weeks old) by topical dinitrochlorobenzene (DNCB) application on shaved backs starting from day 0. In parallel, mice were treated with topical application of STA-9090 (0.5 μmol) in volume of 200 μL dissolved in DMSO:acetone mixture (1:40 vol/vol) or the equivalent volume of DMSO:acetone mixture (vehicle) 5 times a week over a 2-week treatment period starting from day 0. STA-9090 and DNCB were topically administered to mice with 2-h interval. (**b**) Clinical disease severity of vehicle and STA-9090-treated mice was calculated as cumulative SCORAD index at the final day (day 14) of the experiment; corresponding representative clinical presentations of vehicle- and STA-9090-treated mice at the end of the 14-day treatment period are shown on the right. (**c**) IgE levels were measured in sera of vehicle- or STA-9090-treated mice at the end of the experiment by ELISA. (**d**) Filaggrin (*FLG*) expression was examined in skin biopsies of vehicle- or STA-9090-treated mice at the end of the experiment by RT-qPCR. (**e**) Histamine levels were measured in sera of vehicle- or STA-9090-treated mice at the end of the experiment by ELISA. (**f**) Scratching behaviour in vehicle- and STA-9090-treated animals was counted for 10 min at day 13. Histological disease severity shown by scores for (**g**) epidermal thickness, (**h**) dermal leukocyte infiltration and (**i**) mast cells infiltration at the end of the experiment. Representative H&E and toluidine blue (red arrows indicate mast cells) staining of skin biopsies are shown next to the respective graphs. Data are representative of three independent experiments and expressed as mean ± SEM (with individual values) of a total of 8–9 mice per group. ns, not significant. Bars = 100 μm

Female BALB/c mice were purchased from the Tri-City University Animal Facility—Research Service Center (Poland). Induction of AD-like clinical symptoms in 8 weeks-old mice followed a published protocol with minor modifications (Kim et al. 2018; Jang et al. 2020). Briefly, a 6 cm^2^ area on the mouse back was shaved and depilated on day − 1 of the experiment. On day 0 and 3, 200 µL 1% DNCB followed by 200 µL 0.4% DNCB dissolved in an acetone:olive oil mixture (3:1 vol/vol) was topically applied to the shaved back skin every second day starting from day 6 until day 10 and once more on day 13 as was additionally illustrated in Fig. 1 and Fig. 2.

Skin inflammation was evaluated using a modified version of the Scoring Atopic Dermatitis (SCORAD) at day 14, as described previously with some modifications (Kim et al. 2018; Jang et al. 2020). Briefly, erythema, edema, excoriation, and scaling/dryness were each scored independently by two blinded experienced investigators on a scale from 0 to 3: 0, none; 1, mild; 2, moderate; and 3, severe. The scores of these individual aspects of dermatitis were summed up to calculate the cumulative score from 0 to 12. In addition, to quantify the pruritus symptom, incidence of scratching of the body with hind paws of the mice during 10 min of observation at day 13 was recorded.

### Treatment

In this study mice were treated systemically (intraperitoneally) or topically by the Hsp90 inhibitor, as described previously with minor modifications (Tukaj et al. 2014a, 2017). Briefly, 25 mg/kg of STA-9090 (Selleck Chemicals) dissolved in DMSO (Sigma) or the equivalent volume of DMSO (vehicle) was intraperitoneally injected to mice three times a week over a 2-week treatment period starting from day 0. The amount of DMSO used in this study was well tolerated by mice, with no observed toxicity such as weight loss or mortality. In the case of local treatment, STA-9090 was freshly reconstituted in a DMSO:acetone mixture (1:40 vol/vol) to a concentration of 0.5 μmol and applied topically to back skin, 5 times a week over a two week period. Each treatment dose (administrated either systemically or topically) was applied 2 h prior to DNCB application. Blood and skin samples were taken for further experiments at day 14.

### Histopathology

For the analysis of epidermal hyperplasia or dermal leucocyte infiltration, skin samples of the back obtained on the final day of the experiments (day 14) were fixed in 4% (w/v) buffered formalin and embedded in paraffin. 6-μm tissue sections were stained with hematoxylin and eosin (H&E) (Tukaj et al. 2022). To evaluate mast cell infiltration, 0.01% toluidine blue staining was used as described previously (Kim et al. 2018; Jang et al. 2020). Epidermal thickness or number of mast cells in skin sections was calculated under the light microscope at 100 × magnification in 3–6 randomly selected skin areas of each slide. Semiquantitative evaluation of dermal leukocyte infiltration in H&E skin sections was performed by two experienced researchers in a blinded manner, following a scoring scheme (1 = no infiltration, 2 = mild infiltration, 3 = moderate infiltration, 4 = severe infiltration) as described earlier (Ludwig et al. 2005).

### Enzyme-linked immunosorbent assays

Serum levels of IgE and histamine were measured by commercially available ELISA kits (Invitrogen and Abcam, respectively) according to the manufacturer’s protocol.

### RT-qPCR analysis

Total RNA was extracted from frozen mouse dorsal skin by using RNeasy Mini Kit (QIAGEN), according to the manufacturer's protocol. RNA concentration and purity was evaluated by the ratio of absorbance at 260 nm to 280 nm. cDNA was generated from total RNA using QuantiTect Reverse Transcription Kit (QIAGEN). The mRNA levels were assessed using QuantiNova SYBR Green PCR Kit (QIAGEN) in LightCycler­® 480 Instrument II (Roche). Relative expression of *filaggrin* (FLG) was monitored. Here, using BestKeeper software, three reference genes (GAPDH, BACT, and RLP13A) were selected. The following primers were used:

FLG: forward, 5′-ATGTCCGCTCTCCTGGAAAG-3′ and reverse, 5′-TGGATTCTTCAAGACTGCCTGTA-3′;

GAPDH; forward, 5′-CATCACTGCCACCCAGAAGACTG-3′ and reverse, 5′-ATGCCAGTGAGCTTCCCGTTCAG-3′;

BACT Forward, 5′- CTCTTCCAGCCTTCCTTCCT-3′ and reverse, 5′-AGCACTGTGTTGGCGTACAG-3′, and.

RLP13A: forward, 5′-CTGCTCTCAAGGTTGTTCGGCT-3′ and reverse, 5’-CCTTCCGTTTCTCCTCCAGAGT-3’.

The cycling conditions were as follows: 95 °C for 2 min followed by 50 cycles of denaturation at 95 °C for 5 s, annealing at 60 °C for 10 s, with subsequent melting analysis. Each PCR assay was run in duplicate. The relative gene expression levels were analyzed using the 2^−ΔΔCq^ method.

### Statistical analysis

Statistical calculations were performed using GraphPad Prism software (GraphPad, San Diego, CA, USA). Non-normal and normal distributed data were analyzed by Mann–Whitney U test and Student t-test, respectively. P values less than 0.05 were considered as significant.

## Results

### Intraperitoneal STA-9090 treatment reduces disease activity in a DNCB-induced atopic dermatitis mouse model

AD was induced in mice by repetitive topical administration of DNCB as described previously with minor modifications (Kim et al. 2018; Jang et al. 2020) (Fig. 1a). Intraperitoneally administration of STA-9090 (25 mg/kg) significantly reduced the development of characteristic clinical symptoms of AD i.e., erythema, edema, excoriation, and scaling/dryness (modified SCORAD index) (Fig. 1b) and scratching behavior compared with vehicle-treated animals (Fig. 1c). Histologically, epidermal hyperplasia or dermal leukocyte infiltration were significantly milder in STA-9090-treated mice compared with vehicle-treated control animals (Fig. 1d, e), whereas dermal mast cells infiltration (Fig. 1f), skin expression of *FLG* (Fig. 1g), as well as serum levels of histamine (Fig. 1h), or IgE (Fig. 1i) were not altered by the anti-Hsp90 treatment. Intraperitoneally administered STA-9090 was generally well tolerated, except for an observed average weight loss of approximately 5.96%.

### Topical STA-9090 treatment reduces disease activity in a DNCB-induced atopic dermatitis mouse model

We next investigated the effect of topically applied STA-9090 on DNCB-induced AD inflammation (Fig. 2a). Topically applied STA-9090 (0.5 μmol) significantly reduced the development of characteristic clinical symptoms of AD as expressed as modified SCORAD index compared with vehicle-treated animals (Fig. 2b). Interestingly, topical STA-9090 treatment significantly reduced serum levels of IgE (Fig. 2c) and up-regulated *FLG* expression in skin samples (Fig. 2d). Serum levels of histamine, scratching behavior, epidermal hyperplasia, dermal leukocyte infiltration, and mast cells infiltration to the skin, however; were not altered by the inhibitor (Fig. 2e-i). Topical STA-9090 treatment elicited neither skin irritation nor signs of systemic toxicity such as weight loss or death.

## Discussion

We have previously found that Hsps, including Hsp70 and Hsp90 represent important pathophysiological factors and potential treatment targets in autoimmune blistering skin diseases (AIBDs), such as bullous pemphigoid, dermatitis herpetiformis, and epidermolysis bullosa acquisita (EBA), as well as psoriasis (Tukaj et al. 2013, 2021, 2022; Kasperkiewicz et al. 2014). In addition, we have recently found that significantly elevated levels of circulating Hsp90 in AD patients positively correlated with disease activity (SCORAD) (Sitko et al. 2021). The latter observations deserve attention in the study of the role Hsp90 in AD.

AD is a chronic and presently incurable immune-mediated disease. Intense itching, decreased *FLG* expression in epidermis, and increased levels of circulating IgE are common pathophysiological abnormalities in both AD patients and DNCB-induced mouse model of AD (Jang et al. 2020; Langan et al. 2020). Although, there is no specific cure for AD, it can be effectively controlled. Topical tacrolimus (calcineurin inhibitor) and topical corticosteroids belong to the first line drugs offering to the moderate-to-severe AD patients. In some severe cases, phototherapy, systemic immunosuppressors (e.g., cyclosporine A), JAK Inhibitors, and targeted biologics (e.g., dupilumab) are used. Since one of the hallmarks of AD is the elevated level of circulating IgE (typical for approximately 80% of the patients), this molecule has become AD’s potential therapeutic target. For instance, the blockade of free IgE by omalizumab (anti-IgE monoclonal antibody) showed some clinical benefit in AD patients with poor response to traditional therapy (Wang et al. 2016). In addition, clinical evidence suggests that targeting IgE antibodies by immunoadsorption seems to be an effective treatment option for patients severely affected by AD with highly elevated IgE serum levels (Kasperkiewicz et al. 2018). Despite the effectiveness of mentioned-above treatment in some AD patients, the exact role of IgE in the pathophysiology and the severity of AD is not fully understood (Wollenberg et al. 2021). All these drugs and therapies, however; may cause various side effects, therefore, the development of new treatments for AD without or with limited side effects are needed (Ujiie et al. 2022).

Although most of the designed Hsp90 inhibitors targeting the ATP-binding pocket have entered oncological clinical trials, they have not been finally approved by the *Food and Drug Administration* due to toxicity, weak pharmacokinetic profiles, and insufficient clinical effectiveness. A particular obstacle to the use of these classical inhibitors may be the fact that they trigger a survival mechanism in cancer cells related to heat-shock response (HSR) which involves activation of the heat shock factor 1 (Sanchez et al. 2020). On the other hand, numerous preclinical studies using Hsp90 inhibitors, with N-terminal affinity, seem to be promising for the treatment of inflammatory/autoimmune diseases due to the activation of an HRS and attenuation of various pro-inflammatory molecules (Tukaj and Węgrzyn 2016; Tukaj and Sitko 2022). Recently, a proof-of-concept clinical trial study suggested that Hsp90 inhibition by RGRN-305 may represent a new treatment option for treating psoriasis. RGRN-305 demonstrated acceptable safety, although mild to moderate drug-induced skin rash was reported in some patients (Bregnhøj et al. 2022).

In this study, we demonstrated that ganetespib (STA-9090), a non-benzoquinone ansamycin Hsp90 inhibitor specifically targeting ATP-binding domain, significantly attenuated the development of the disease in mice with AD induced by topical application of DNCB. Although both routes of administration of this inhibitor (i.e., intraperitoneal, or topical) to AD mice resulted in suppression of disease activity as measured by the modified SCORAD index, the effect of this therapy on AD-specific cells and molecules is somewhat different. For instance, while intraperitoneal application of the STA-9090 reduced epidermal hyperplasia and incidence of scratching behavior, topically applied inhibitor had no such effects. On the other hand, only topically applied inhibitor reduced serum levels of IgE and upregulated the expression of *FLG.* Mentioned-above discrepancies, despite similar clinical effectiveness, resulted from the route of administration of the Hsp90 inhibitor to mice with AD. Several issues concerning e.g., absorption, distribution, metabolism, and elimination of the drug delivered either locally or systemically ultimately affect the target cells involved in the development of the disease. It is also noteworthy that despite the local induction of the disease, there are many pathological changes found in the periphery. Therefore, due to the heterogeneous character of AD (van der Schaft et al. 2019), potential use of anti-Hsp90 therapy (locally or peripherally) along with other drugs in AD should be personalized in the future.

Our results are in line with previously published observations concerning amelioration of other inflammatory skin condition such as EBA. We have proven that either systemically or topically applied Hsp90 inhibitors belonging to the geldanamycin derivatives (i.e., 17-DMAG or 17AAG) or short peptide derivative TCBL-145 ameliorated disease activity in experimental mouse models of EBA. The mentioned-above therapy was associated with e.g., reduced neutrophilic infiltrates, key effector cells in the pathogenesis of EBA (Kasperkiewicz et al. 2011; Tukaj et al. 2014a, 2017).

It is generally assumed that mast cells contribute to skin inflammation in AD and are mainly responsible for i.e., histamine secretion (Kawakami et al. 2009). In this study, we found no effects of the treatment on mast cell infiltration and histamine liberation. Since lesioned skin of patients with AD shows infiltration of various immune cells, including T lymphocytes, mainly belonging to the Th2 subtypes, but also Th1 and Th17 (Weidinger and Novak 2016; Langan et al. 2020), we can assume that the reduced skin infiltration observed in STA-9090-treated mice was due to the sensitivity of immune cells other than mast cells in response to the therapy. Based on our previous in vitro and pre-clinical observations, we can speculate that T lymphocytes belonging to Th1 or Th17 subpopulations (Kasperkiewicz et al. 2011; Tukaj et al. 2014b), might represent such candidates. It should also be considered that the inflammatory profile in the skin of AD patients is complex and diverse, with activation of skin-resident inflammatory cells such as e.g., dendritic cells, innate lymphoid cells, or Langerhans cells (Weidinger and Novak 2016; Langan et al. 2020). Therefore, the immunosuppressive mechanism of action of Hsp90 inhibition in alleviating AD needs to be further elucidated.

In this study, we showed that systemic or local inhibition of Hsp90 activity significantly attenuated clinical features in AD mice. Our results suggest that topical delivery of STA-9090 offers the benefit of a reduced risk of systemic adverse effects of Hsp90 inhibition. It is especially important as toxicity of these drugs has been frequently observed after systemic administration in oncological clinical trials (Sanchez et al. 2020). Although topical STA-9090 treatment elicited neither skin irritation nor signs of systemic toxicity such as weight loss or death in mice with AD, long-term safety studies of topical Hsp90 inhibitors are still needed.

## Conclusion

In conclusion, systemic blockade of Hsp90 activity by STA-9090 significantly alleviated skin inflammation in mice with AD, as evidenced by lower disease activity, reduced epidermal hyperplasia or dermal leukocyte infiltration, and limited pruritus in animals. In addition, topically applied STA-9090 was also effective in ameliorating disease symptoms that was paralleled with reduced circulating IgE secretion and upregulated *FLG* expression in AD mice. Taken together, our results suggest that the topical application of Hsp90 inhibitors offers greater therapeutic benefits due to a reduced risk of systemic side effects and therefore may be useful for controlling AD and possibly other related inflammatory skin diseases.
